# Supplementary material for: Dbf4-dependent kinase finetunes Ino80 function at chromosome replication origins
Source: Nat Commun. 2026 Mar 28;17:3029. doi: 10.1038/s41467-026-70698-4 (PMC13035910; doi:10.1038/s41467-026-70698-4)
Supplement: Supplementary file 1 — Supplementary Information [file 41467_2026_70698_MOESM1_ESM.pdf]

Supplementary Information

Dbf4-Dependent Kinase Finetunes INO80 Function at Chromosome Replication Origins

Priyanka Bansal [1*], Shibojyoti Lahiri [1*], Chandni Natalia Kumar [1], Jessica Furtmeier [1], Lorenz Spechtenhauser [1], Lorenzo Galanti [2,3], Juan de Dios Barba Tena [2], Erika Chacin[1,4], Garp Linder [5], María Ángeles Ortíz-Bazán [6], Marisa Müller [1], Petra Vizjak [7,8], Tobias Straub [1], Felix Mueller-Planitz [7], Johannes Stigler [5], Andrés Aguilera [6], Belen Gómez-González [6], Boris Pfander [2], Philipp Korber [1], Axel Imhof [1#] and Christoph F. Kurat [1,9#]

Affiliations

[1] Biomedical Center Munich (BMC), Division of Molecular Biology, Faculty of Medicine, Ludwig-Maximilians-Universität in Munich, Martinsried, Germany

[2] Cell Biology, Dortmund Life Science Center (DOLCE), TU Dortmund University, Department of Chemistry and Chemical Biology, Dortmund, Germany

[3] Present address: DSB Repair Metabolism Laboratory, The Francis Crick Institute, London, UK

[4] Present address: Bayer AG, Pharmaceuticals, Research and Development, Genomic Medicine, Aprather Weg 18a, 42113 Wuppertal - Aprath, Germany

[5] Gene Center and Department of Biochemistry, Ludwig-Maximilians-Universität in Munich, Martinsried, Germany

[6] Centro Andaluz de Biología Molecular y Medicina Regenerativa-CABIMER, Universidad de Sevilla-CSIC, Seville, Spain

[7] Institute of Physiological Chemistry, Faculty of Medicine Carl Gustav Carus, Technische Universität Dresden, Dresden, Germany

[8] Present address: Early-Stage Bioprocess Development, Boehringer Ingelheim Pharma GmbH & Co. KG, Biberach an der Riss, Germany Gene Center and Department of Biochemistry, Ludwig-Maximilians-Universität in Munich, Martinsried, Germany

[9] Present address: Chair of Genetics, University of Bayreuth, Bayreuth, Germany

*These authors contributed equally to this work.

[#] To whom correspondence should be addressed:

C.F.K.: Christoph.kurat@bmc.med.lmu.de

A.I.: Imhof@lmu.de

Supplementary Figure 1

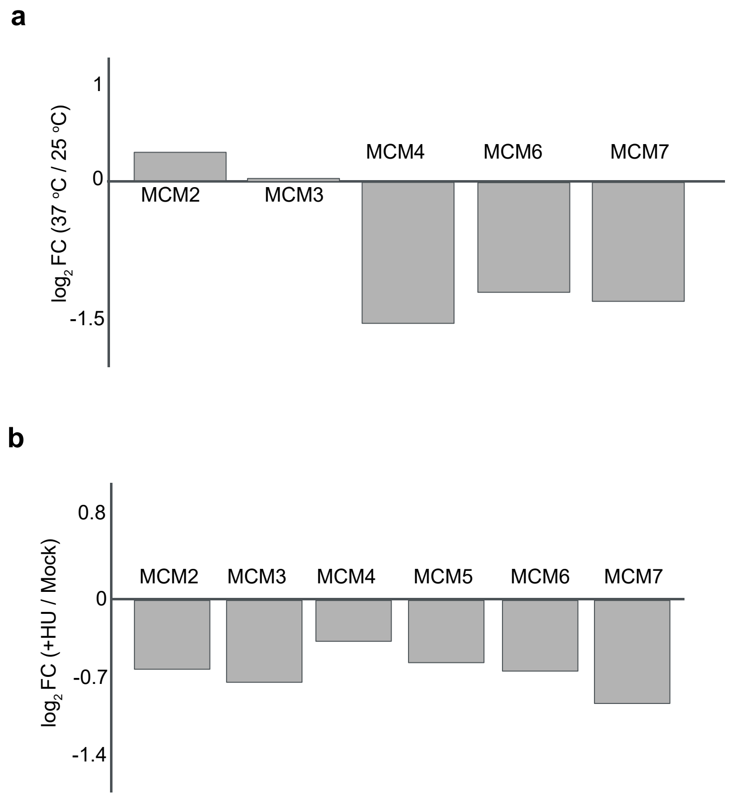

Supplementary Figure 1: MCM complex subunits found in phospho-proteomic network.

- **a.** MCM complex subunits found in the *cdc7-4* screen.
- **b.** MCM complex subunits found in the HU screen.

Supplementary Figure 2

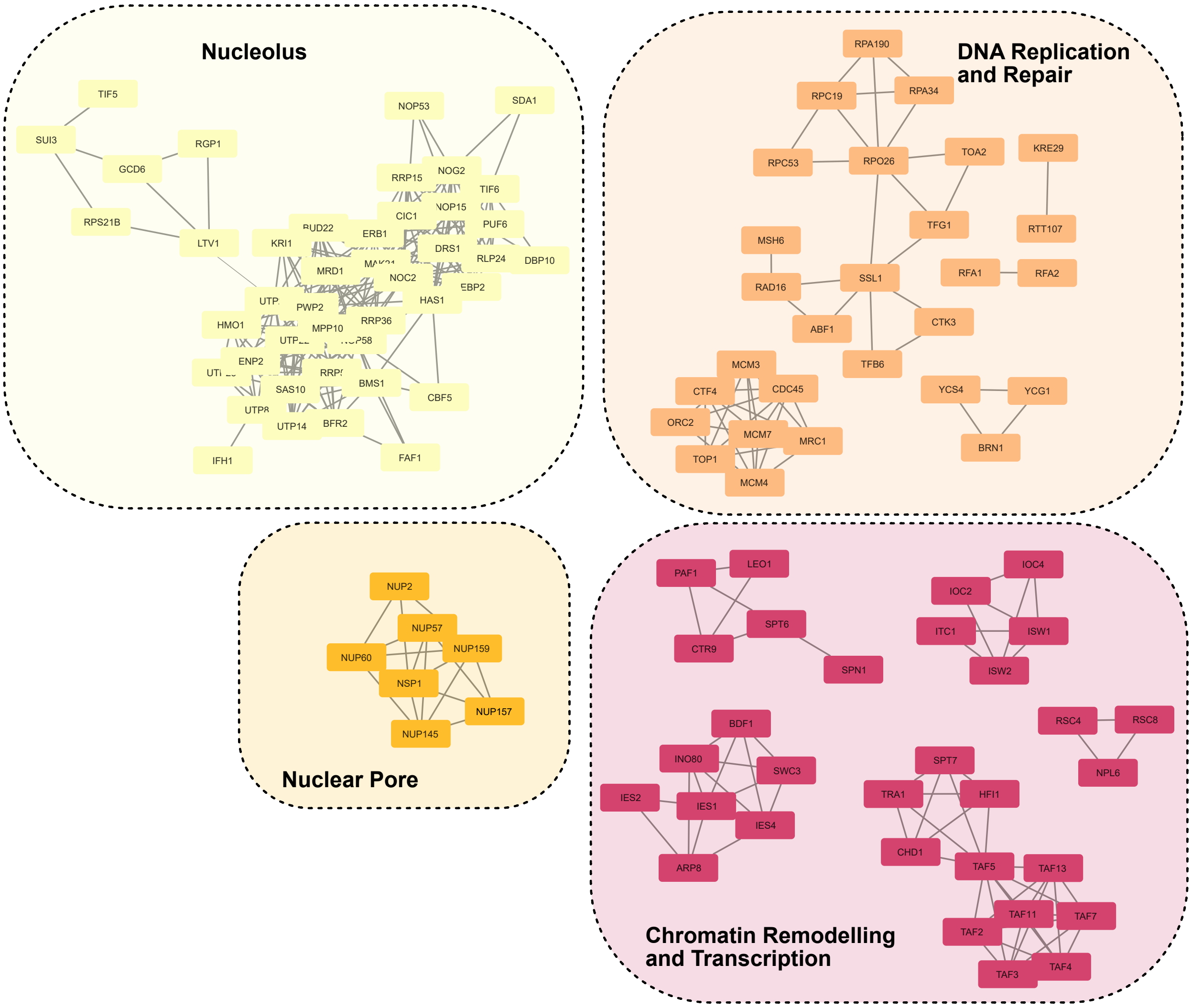

Supplementary Figure 2:

Network of phosphorylation targets influenced by DDK-inhibition involved in DNA replication and repair or chromatin remodeling and transcription.

The protein-protein interaction network was visualized using Cytoscape[1] (vs 3.10.0). For network analysis those proteins were considered, which are known to be localized to the nucleus and for which we detected a clear increase in phosphorylation at the permissive temperature or without HU. Interaction data were taken from the STRING database[2].

Supplementary Figure 3

a

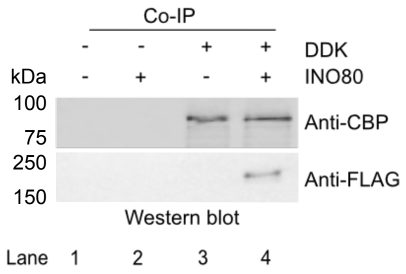

b

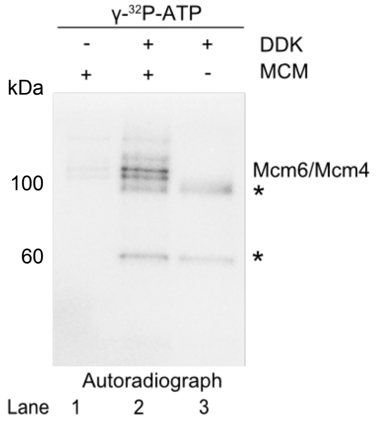

Supplementary Figure 3: INO80 is a bona-fide target of DDK.

- **a.** *In vitro* Co-Immunoprecipitations assay with INO80 and DDK. N=2.
- **b.** Incorporation of [^{32}P]-γ-ATP into MCM by DDK as in Fig. 2 d. The presence of asterisks indicates the occurrence of auto-phosphorylation of DDK, as determined through a reaction with DDK alone (Fig. 2d, Lane 1). N=2. Source data are provided as a Source Data File.

Supplementary Figure 4

a

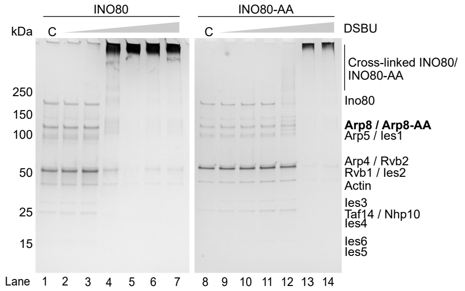

b

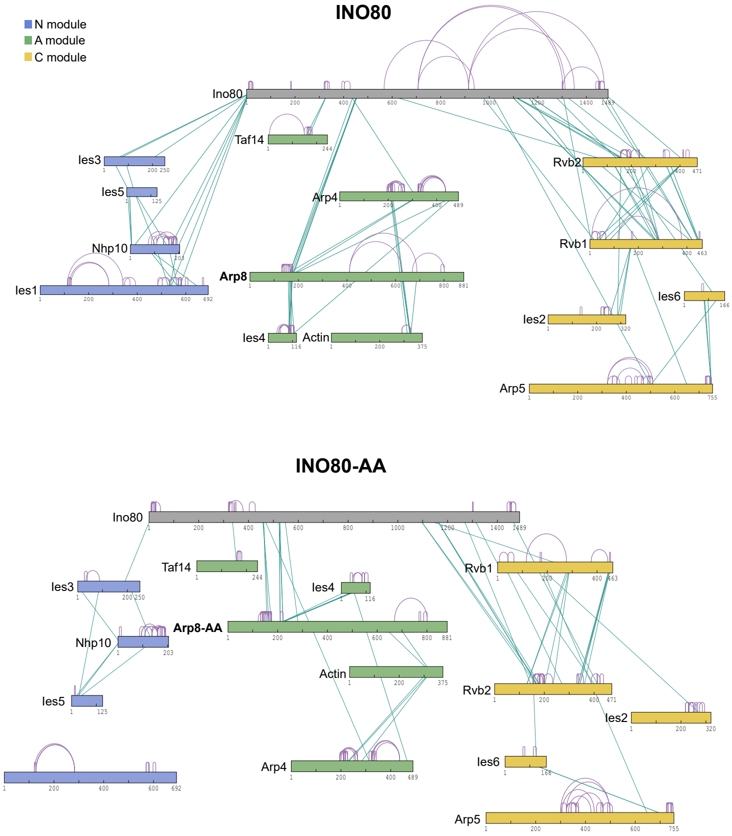

Supplementary Figure 4: Subunit interaction topology maps of INO80 and INO80-AA complexes.

a. SDS-PAGE and silver stain analysis of the DSBU crosslinker titration with INO80 and INO80-AA complexes. Complexes were tested with a range of 25 – 208 µM DSBU as represented by grey triangle on the top. C refers to control reaction without any crosslinker and kDa refers to the size of proteins in kilo-daltons.

b. Crosslink network map of INO80 and INO80-AA complexes. Different subunits and modules of INO80 are color-coded in accordance with (Fig. 2e). Inter cross-links are represented in green and intra cross-links are represented in purple within the complex. Source data are provided as a Source Data File.

Supplementary Figure 5

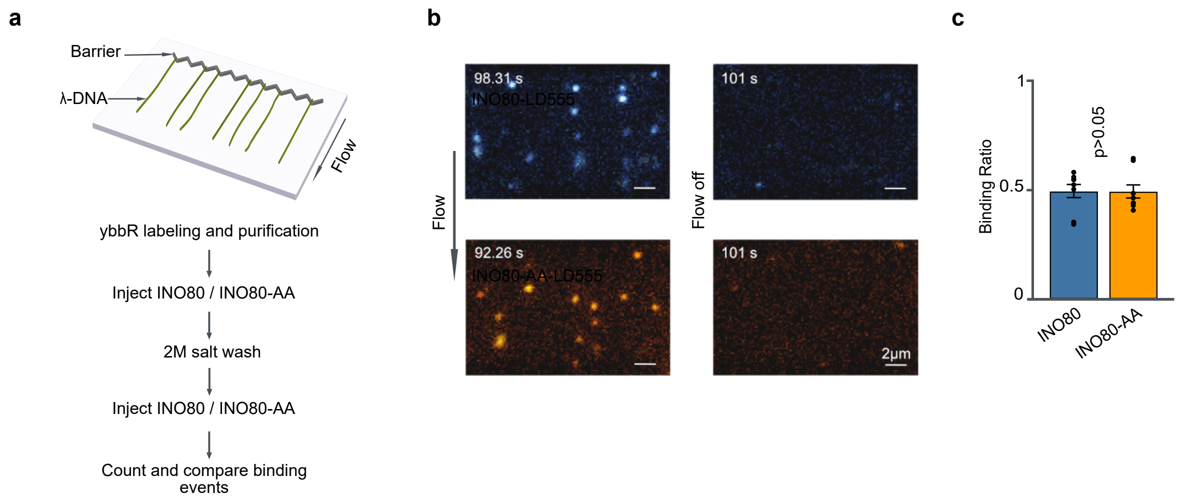

Supplementary Figure 5: Interaction of INO80 and INO80-AA with DNA.

- **a.** Schematic of a single tethered DNA curtain under flow. Below schematic is the outline of the assay to measure the binding affinity of labelled and purified INO80 and INO80-AA complexes to lambda (λ) DNA.
- **b.** Representative wide-field images of INO80 (top left) and INO80-AA (bottom left) complexes bound to flow stretched λ DNA. After stopping the flow, λ DNA molecules bound to INO80 (top right) and INO80-AA (bottom right) complexes move out of the field view.
- **c.** The graph shows mean ratios of INO80 to INO80-AA binding events to the λ DNA for 8 double injections respectively. To remove binding bias of the complexes, they were injected, followed by a salt wash and then INO80-AA or vice versa. For each injection, five averaged frames were analyzed and in total 3317 binding events were counted. Acquired ratios were bootstrapped 10000 times and error bars represent the one sigma limits of the resampled means. The p-value was obtained by using two-tailed unpaired t-test calculations. Source data are provided as a Source Data File.

Supplementary Figure 6

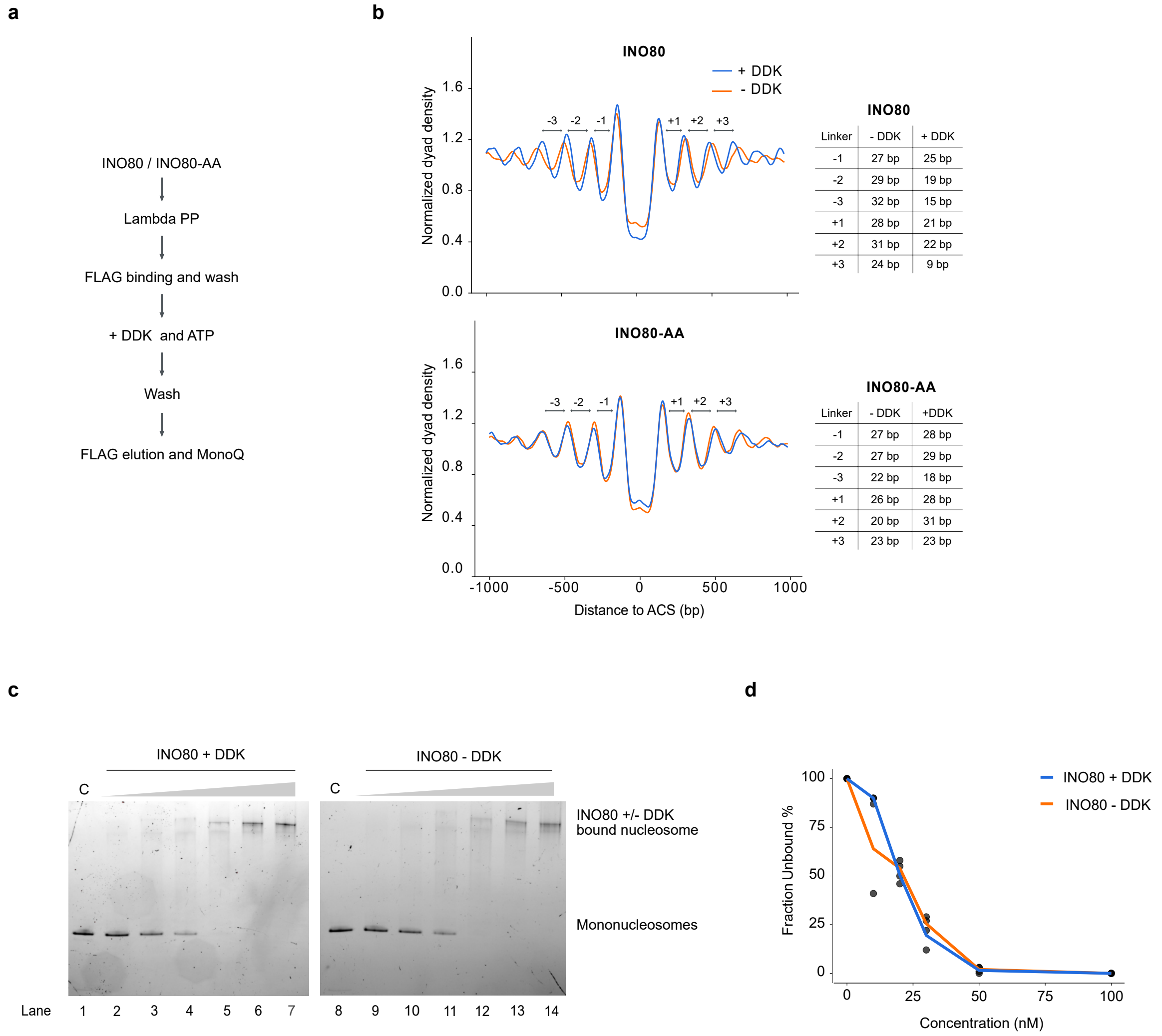

Supplementary Figure 6: The dependence of INO80's chromatin function on DDK-dependent in vitro phosphorylation.

- **a.** Purified INO80 and INO80-AA complexes were dephosphorylated using Lambda Phosphatase (Lambda PP) and re-phosphorylated with purified DDK.
- **b.** Composite plot as in (B) illustrating representative *in vitro* MNase-seq data for SGD chromatin incubated with ORC plus either DDK phosphorylated (blue) or dephosphorylated (orange) INO80 and INO80-AA complexes. N=2.
- **c.** The binding of dephosphorylated INO80 plus/minus DDK to mononucleosomes was assessed using an electrophoretic mobility shift assay (EMSA) coupled with native polyacrylamide gel electrophoresis (PAGE) as in Fig. 3d. N = 2.
- **d.** The unbound fractions (mononucleosomes) in (c) were quantified for all experiments and plotted as in Fig. 3d. The results of the two experiments are presented as individual data points. Source data are provided as a Source Data File.

Supplementary Figure 7

Replicate 1

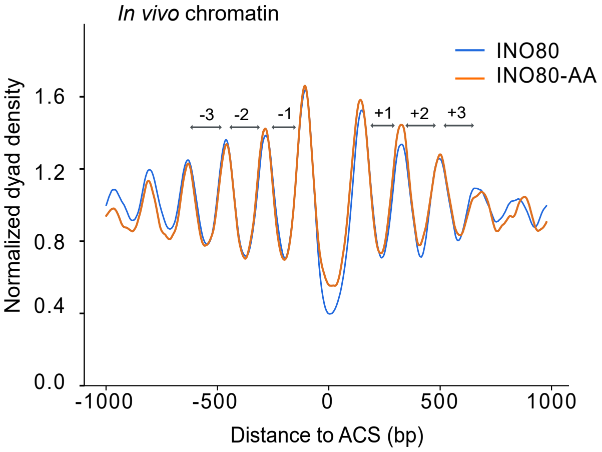

Linker	INO80	INO80-AA
-1	30 bp	29 bp
-2	31 bp	30 bp
-3	24 bp	25 bp
+1	33 bp	33 bp
+2	22 bp	24 bp
+3	22 bp	35 bp

Replicate 2

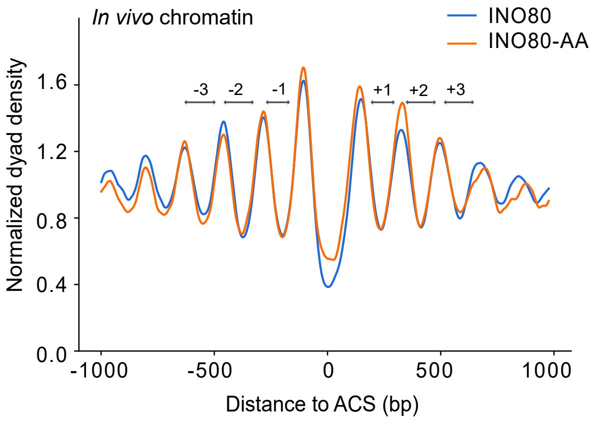

Linker	INO80	INO80-AA
-1	30 bp	30 bp
-2	30 bp	28 bp
-3	24 bp	25 bp
+1	32 bp	40 bp
+2	22 bp	21 bp
+3	21 bp	46 bp

Supplementary Figure 7: *In vivo* MNase profile of wild type and *arp8-AA* mutant. Composite plot representations as in (Fig. 4b) illustrating *in vivo* MNase-seq for wild type versus *arp8-AA*. The accompanying table presents the linker lengths of the first, second, third linkers (DNA between nucleosomes) upstream and downstream of NFR (Nucleosome Free Region). Both biological replicates are shown. N=2. Source data are provided as a Source Data File.

Supplementary Figure 8

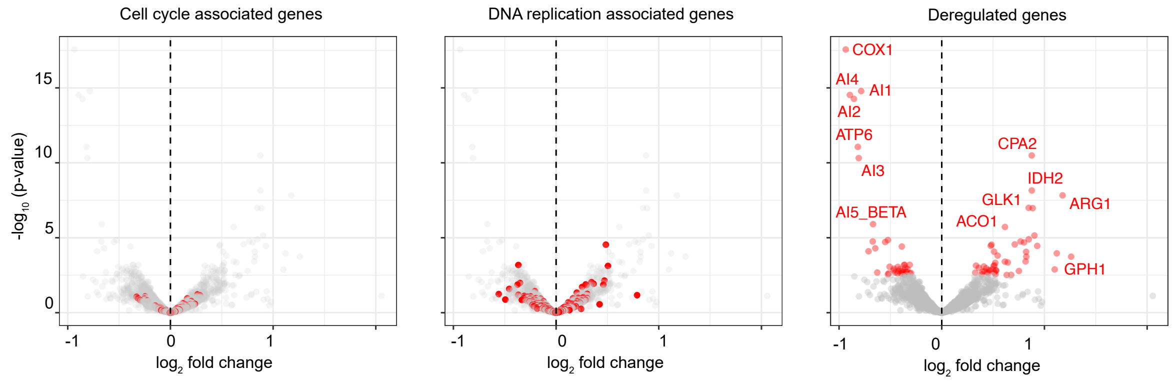

Supplementary Figure 8:
Transcriptional responses of *arp8-AA* compared to *ARP8* in cells grown asynchronously.

Volcano plots showing differential expression of *arp8-AA* versus *ARP8*. The red dots represent genes involved in the cell cycle (left panel, see Supplementary Data 1), replication (middle panel, see Supplementary Data 1), or significantly deregulated genes (right panel). The $\log_2$ fold change shows the extent to which gene expression is either upregulated or downregulated in *arp8-AA*. N = 3.

Supplementary References

1. Shannon, P. et al. Cytoscape: a software environment for integrated models of biomolecular interaction networks. *Genome Res* **13**, 2498-504 (2003).
2. Szklarczyk, D. et al. The STRING database in 2023: protein-protein association networks and functional enrichment analyses for any sequenced genome of interest. *Nucleic Acids Res* **51**, D638-d646 (2023).
3. Winston, F., Dollard, C. & Ricupero-Hovasse, S.L. Construction of a set of convenient Saccharomyces cerevisiae strains that are isogenic to S288C. *Yeast* **11**, 53-5 (1995).
4. Brachmann, C.B. et al. Designer deletion strains derived from Saccharomyces cerevisiae S288C: a useful set of strains and plasmids for PCR-mediated gene disruption and other applications. *Yeast* **14**, 115-32 (1998).
5. On, K.F., Beuron, F., Frith, D., Snijders, A.P., Morris, E.P. & Diffley, J.F. Prereplicative complexes assembled in vitro support origin-dependent and independent DNA replication. *Embo j* **33**, 605-20 (2014).

Supplementary Table 1: *S. cerevisiae strains used in this study*

Name	Genotype	Source
yCFK 51	MAT a, *his3Δ1 leu2Δ0 met15Δ0 ura3Δ0*	[3,4]
yCFK43	MAT α, *his3Δ1 leu2Δ0 lys2Δ0 ura3Δ0*	[3,4]
yPB3	MAT α, *his3Δ1 leu2Δ0 lys2Δ0 ura3Δ0 ARP8::ARP8-S65A, S233A*	This study
yPB4	MAT a, *his3Δ1 leu2Δ0 ura3Δ0 ARP8::ARP8-S65A, S233A*	This study
yPB5	MAT α, *his3Δ1 leu2Δ0 lys2Δ0 ura3Δ0 ARP8::ARP8-S65A, S233A, Ino80-3x FLAG::NAT*	This study
yPB6	MAT a, *his3Δ1 leu2Δ0 ura3Δ0 ARP8::ARP8-S65A, S233A, Ino80-3x FLAG::NAT*	This study
yPB7	MAT a, *his3Δ1 leu2Δ0 met15Δ0 ura3Δ0, Ino80-3x FLAG::NAT*	This study
yCFK23	MAT a, *ade2-1 ura3-1 his3-11,15 trp1-1 leu2-3,112 can1-100, cdc7-4, pep4:: Hyg, his3::HIS3pRS303/SLD3-13MYC, trp1::TRP1pRS304/SLD2, leu2::LEU2pRS305/ SLD7, CDC45, ura3::URA3pRS306/ DPB11*	[5]
yPB8	MAT α, *his3Δ1 leu2Δ0 lys2Δ0 ura3Δ0 ARP8::ARP8-S65A, S233A, Ino80-ybbR-3x FLAG::NAT*	This study
yPB9	MAT a, *his3Δ1 leu2Δ0 met15Δ0 ura3Δ0, Ino80-ybbR-3x FLAG::NAT*	This study

Supplementary Table 2: *Oligonucleotides used in this study*

Name	5'-3' Sequence	Purpose	Purification
oPB1	TAAATTACTAGTCAATAGTACATA AATACAGGGATACAATCGCACCTA ACCGGATCCCCGGGTTAATTAA	*URA* integration at *ARP8* locus F	Desalt
oPB2	TGCAAAGACCTTTCAGAAAAAAAG ATAACAAAAACTTCCATATGCATA TCGAATTCGAGCTCGTTTAAAC	*URA* integration at *ARP8* locus R	Desalt
oPB3	CGTAGTTGGATCTGAGACACCCAG AGCTGTAACAGGTCTTTCTGTTGAC CCA	*ARP8* Serine 65 SDM F	Desalt
oPB4	TGGGTCAACAGAAAGACCTGTTAC AGCTCTGGGTGTCTCAGATCCAAC TACG	*ARP8* Serine 65 SDM R	Desalt
oPB5	GAGAAATAACAACACTAGCCAAAT AGCTAGTACCAACACACCAGATGT TAT	*ARP8* Serine 233 SDM F	Desalt

oPB6	ATAACATCTGGTGTGTTGGTACTA GCTATTTGGCTAGTGTTGTTATTTC TC	*ARP8* Serine 233 SDM R	Desalt
oPB7	TAAATTACTAGTCAATAGTACATA AATACAGGGATACAATCGCACCTA ACATGTCGCAAGAAGAAGCAG	*arp8* integration at *ARP8* locus F	Desalt
oPB8	TGCAAAGACCTTTCAGAAAAAAAG ATAACAAAAACTTCCATATGCATA TCCTAGTACGTGAAAATACATTTA TATTGTAAG	*arp8* integration at *ARP8* locus R	Desalt
oPB9	CCGTGCAAGATGACTTATTT	*ARP8* locus integration check F	Desalt
oPB10	AGCCGTCTAAGCGGGACTAT	*ARP8* locus integration check F	Desalt
oPB11	TGAGGAACCCATTGATATTCCG	*ARP8* S65 sequencing F	Desalt
oPB12	GTCTGCACAGCAGGTGGTGCCCT	*ARP8* S65 sequencing R	Desalt
oPB13	AGACTAGGACGTATTAAAGCT	*ARP8* S233 sequencing F	Desalt
oPB14	GATCCAGGATGTATCACGATA	*ARP8* S233 sequencing R	Desalt
oPB15	GACCATCCTGTCGTAGTTCCAA	*ARP8* sequencing F	Desalt
oPB16	GCAAAAGCATAAGTCAAGATGGA ATTAAGGAAGCGGCAAGTGCATTG GCACGTACGCTGCAGGTCGAC	*INO80* C-terminus 3xFLAG F	Desalt
oPB17	GATAGACATTAACTCCGCTTAATG TAAATAACACAATATGAATACCTT TTATCGATGAATTCGAGCTCG	*INO80* C terminus 3xFLAG R	Desalt
oPB18	ATCAACTTGAAAATTTGTGGGA	*ARP8* sequencing R	Desalt

Supplementary Table 3: *Oligonucleotides for the nucleosome preparation and Windom 601 DNA sequence*

Nucleosome-0N80-fwd	CTGGAGAATCCCGGTGCCGAGG
Nucleosome-0N80-rev	CGGTACCCGGGGATCCTCTAG
Windom 601 DNA sequence (5' FAM)	CTGGAGAATCCCGGTGCCGAGGCCGCTCAATTGGT CGTAGCAAGCTCTAGCACCGCTTAAACGCACGTAC GCGCTGTCCCCCGCGTTTTAACCGCCAAGGGGATT ACTCCCTAGTCTCCAGGCACGTGTCAGATATATAC ATCCTGTGCATGTATTGAACAGCGACCTTGCCGGT GCCAGTCGGATAGTGTTCCGAGCTCCCACTCTAGA GGATCCCCGGGTACCGA
