# Supplementary material for: Dbf4-dependent kinase finetunes Ino80 function at chromosome replication origins
Source: Nat Commun. 2026 Mar 28;17:3029. doi: 10.1038/s41467-026-70698-4 (PMC13035910; doi:10.1038/s41467-026-70698-4)
Supplement: Supplementary file 2 — Description of Additional Supplementary Files [file 41467_2026_70698_MOESM2_ESM.pdf]

Description of Additional Supplementary Files

Supplementary Data 1. Lists the genes included in our analysis to assess transcriptional responses of replication- and cell cycle–related genes, respectively.
