# Supplementary figures and images for: Dbf4-dependent kinase finetunes Ino80 function at chromosome replication origins
Source: Nat Commun. 2026 Mar 28;17:3029. doi: 10.1038/s41467-026-70698-4 (PMC13035910; doi:10.1038/s41467-026-70698-4)

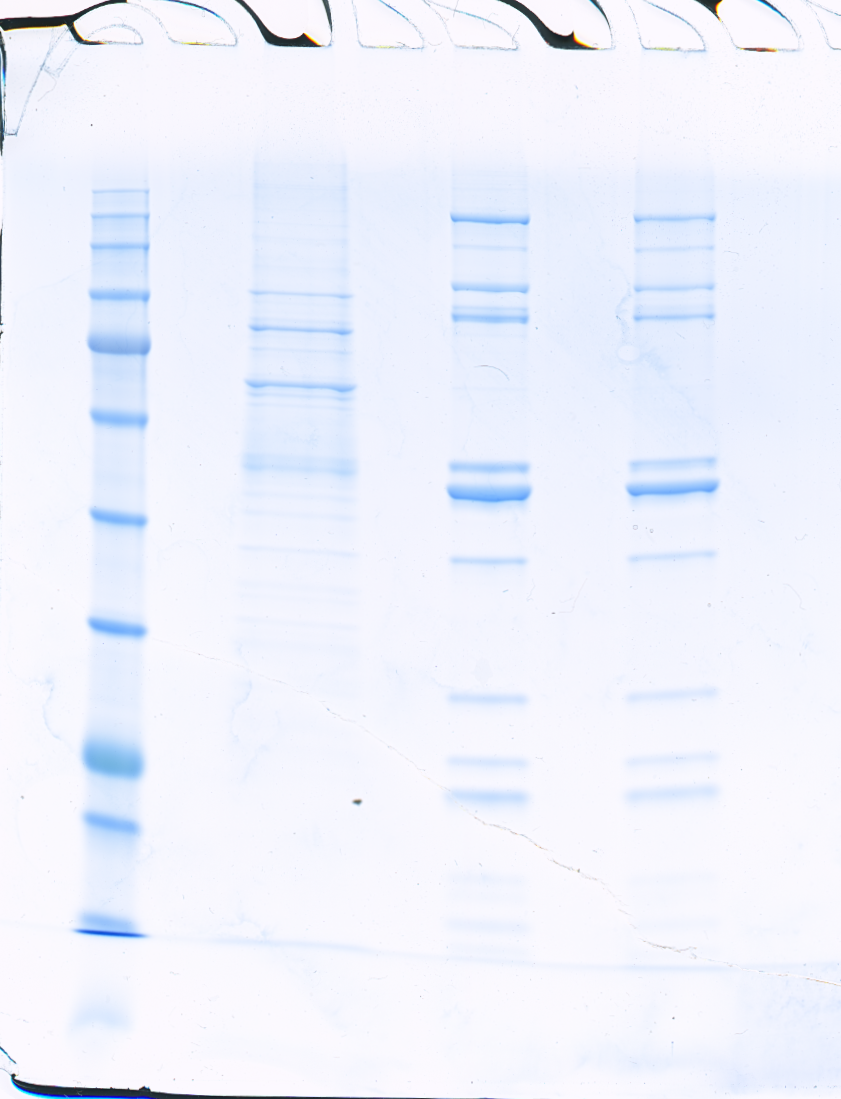

Supplement: Supplementary file 6 — Source data [file 41467_2026_70698_MOESM6_ESM.zip › Uncropped_images/2b.tif]

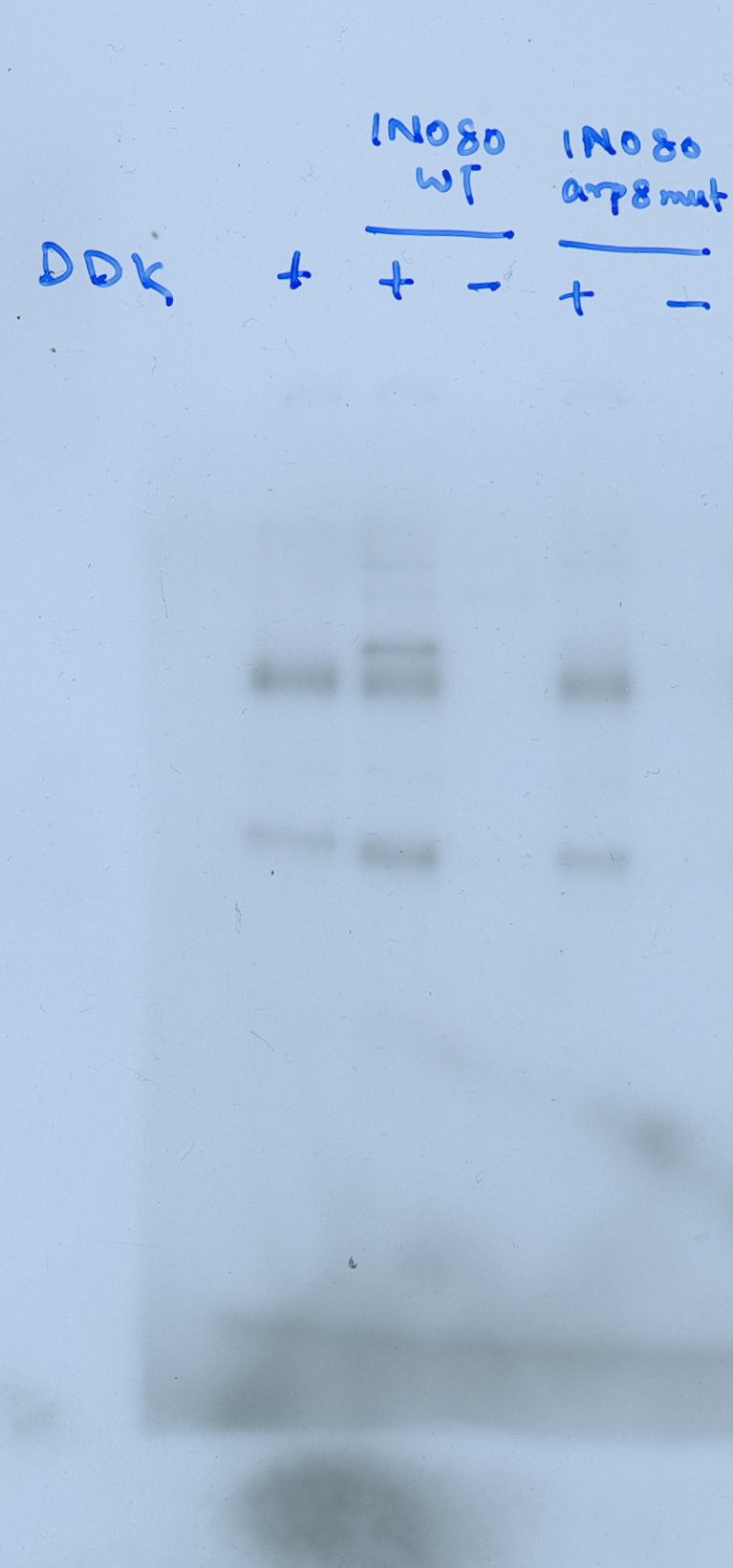

Supplement: Supplementary file 6 — Source data [file 41467_2026_70698_MOESM6_ESM.zip › Uncropped_images/2d.tif]

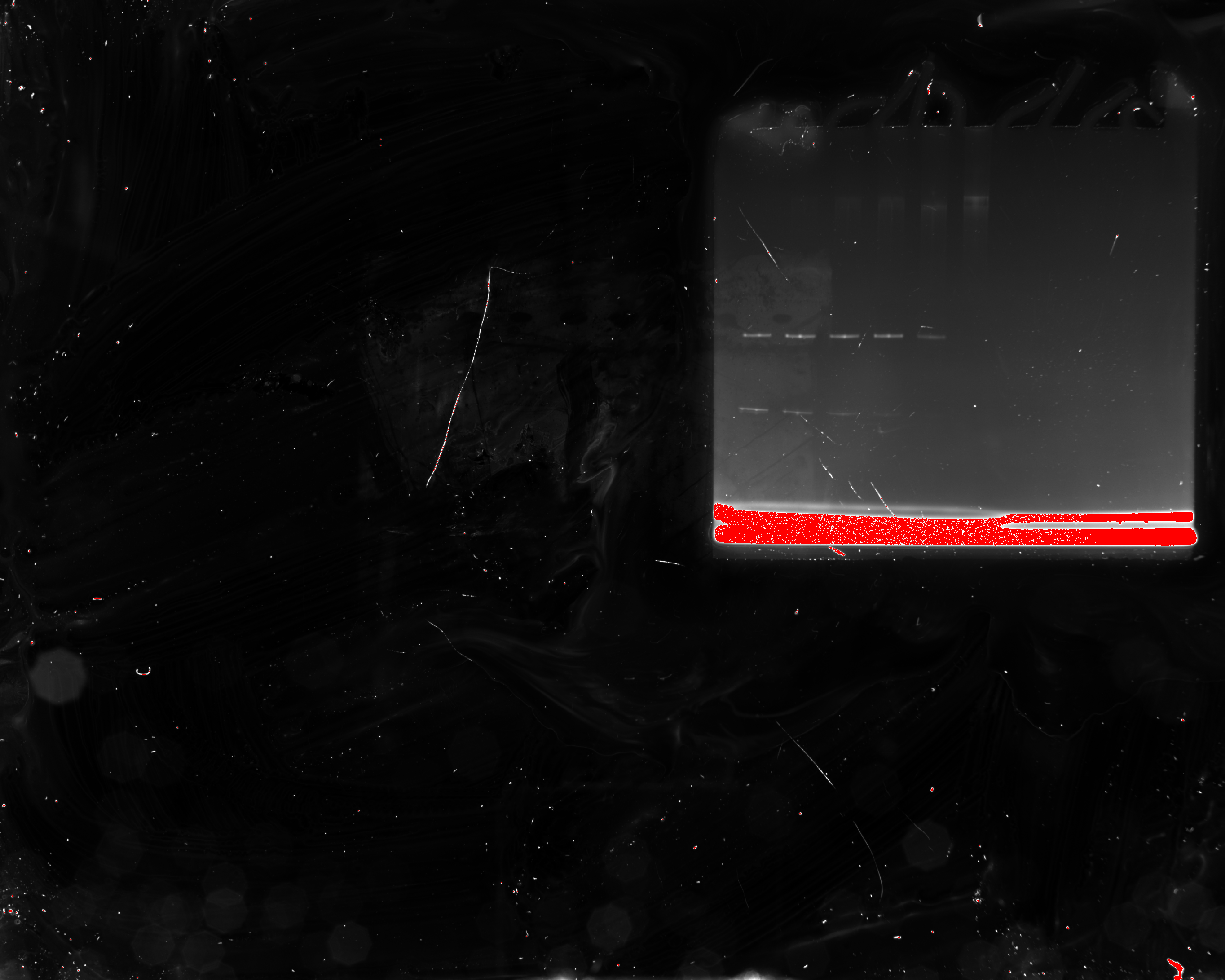

Supplement: Supplementary file 6 — Source data [file 41467_2026_70698_MOESM6_ESM.zip › Uncropped_images/3d.1.tif]

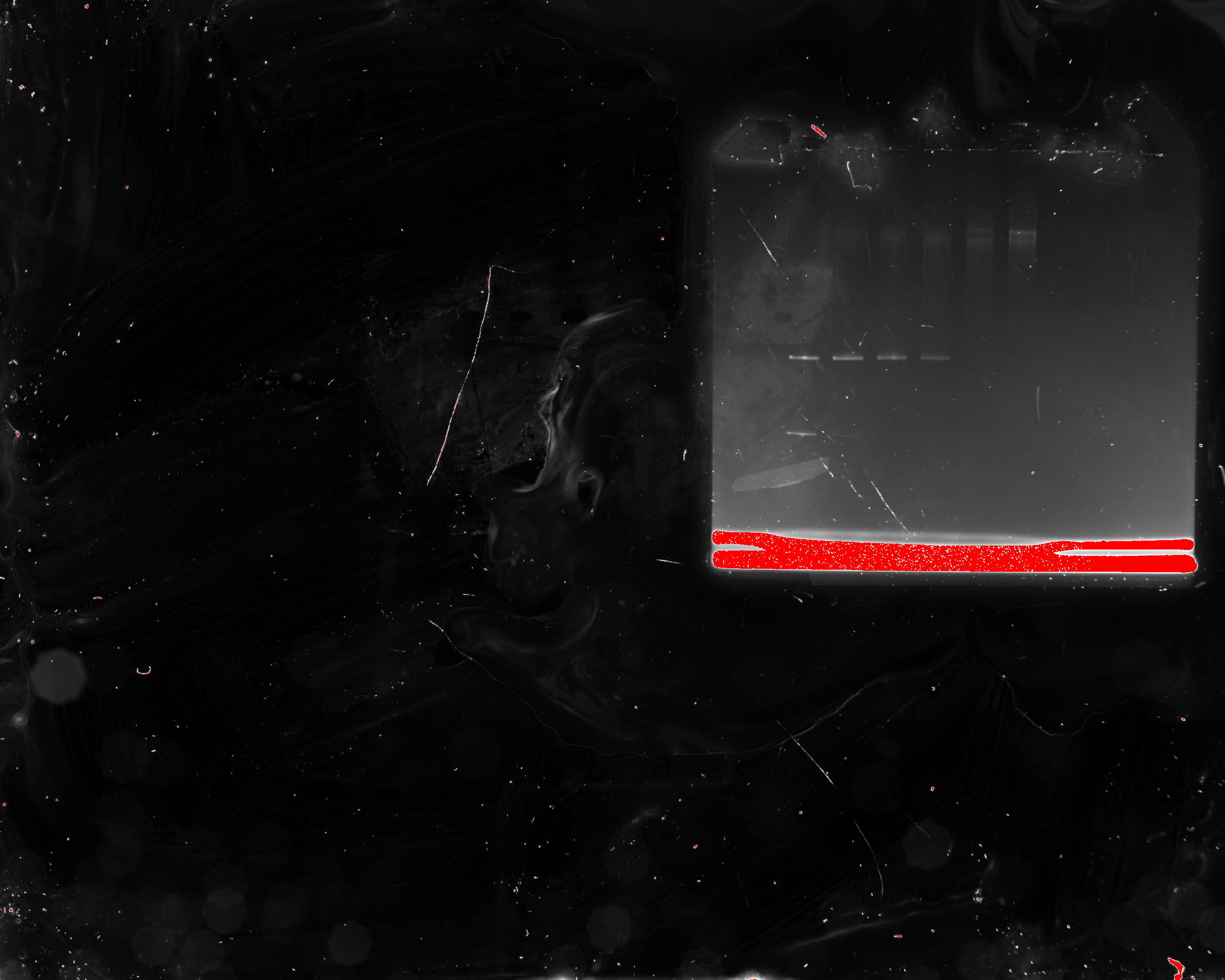

Supplement: Supplementary file 6 — Source data [file 41467_2026_70698_MOESM6_ESM.zip › Uncropped_images/3d.2.tif]

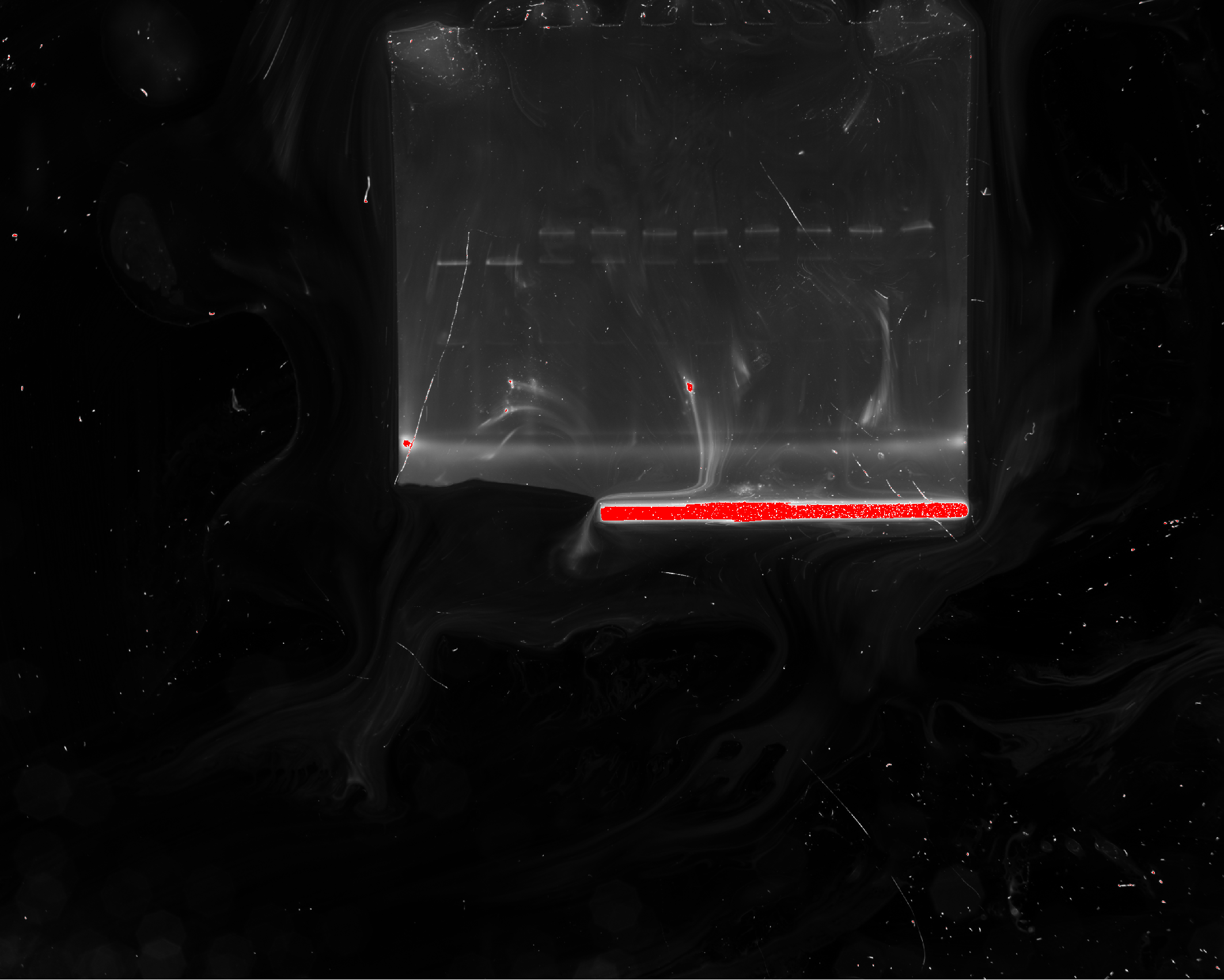

Supplement: Supplementary file 6 — Source data [file 41467_2026_70698_MOESM6_ESM.zip › Uncropped_images/3f.1.tif]

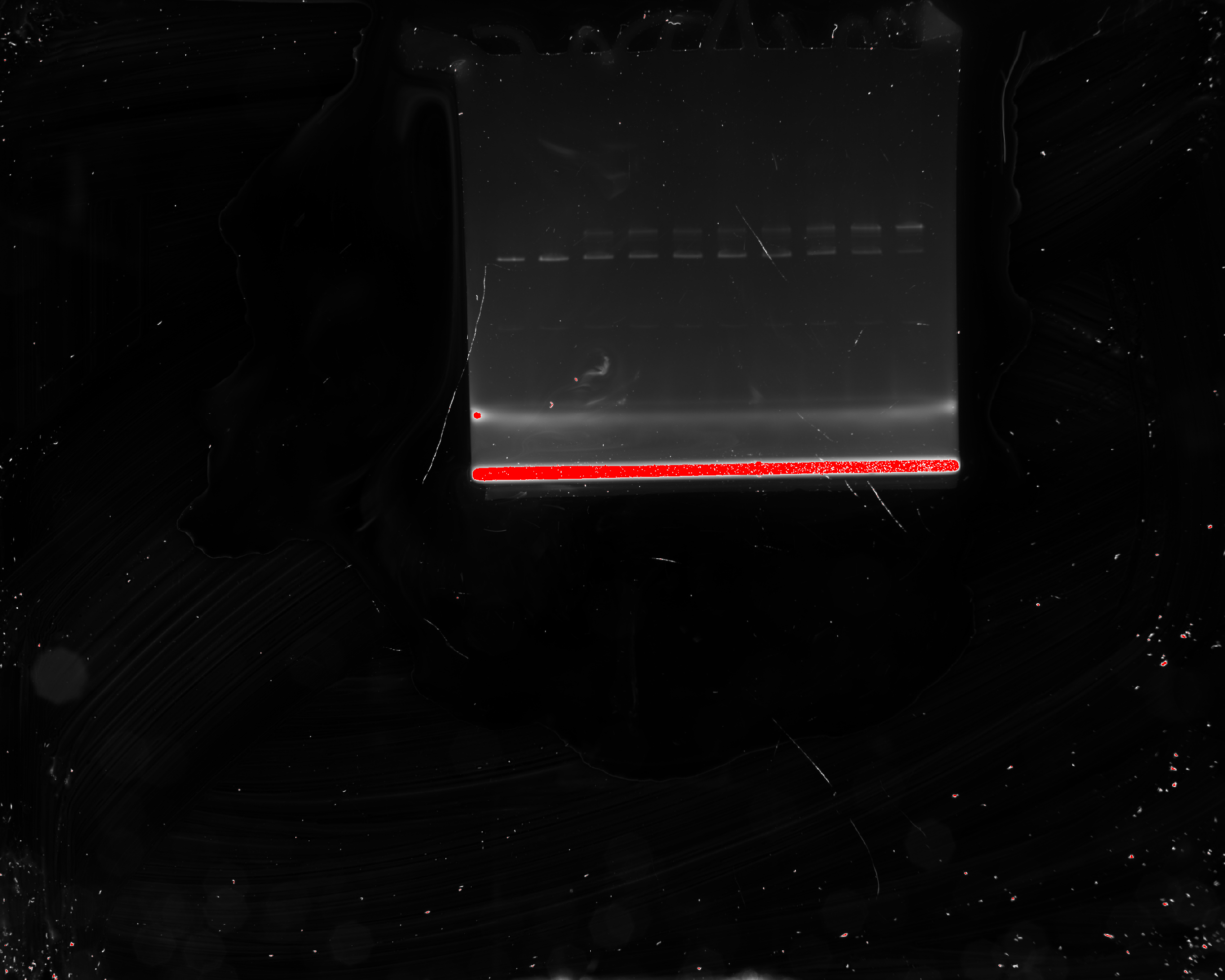

Supplement: Supplementary file 6 — Source data [file 41467_2026_70698_MOESM6_ESM.zip › Uncropped_images/3f.2.tif]

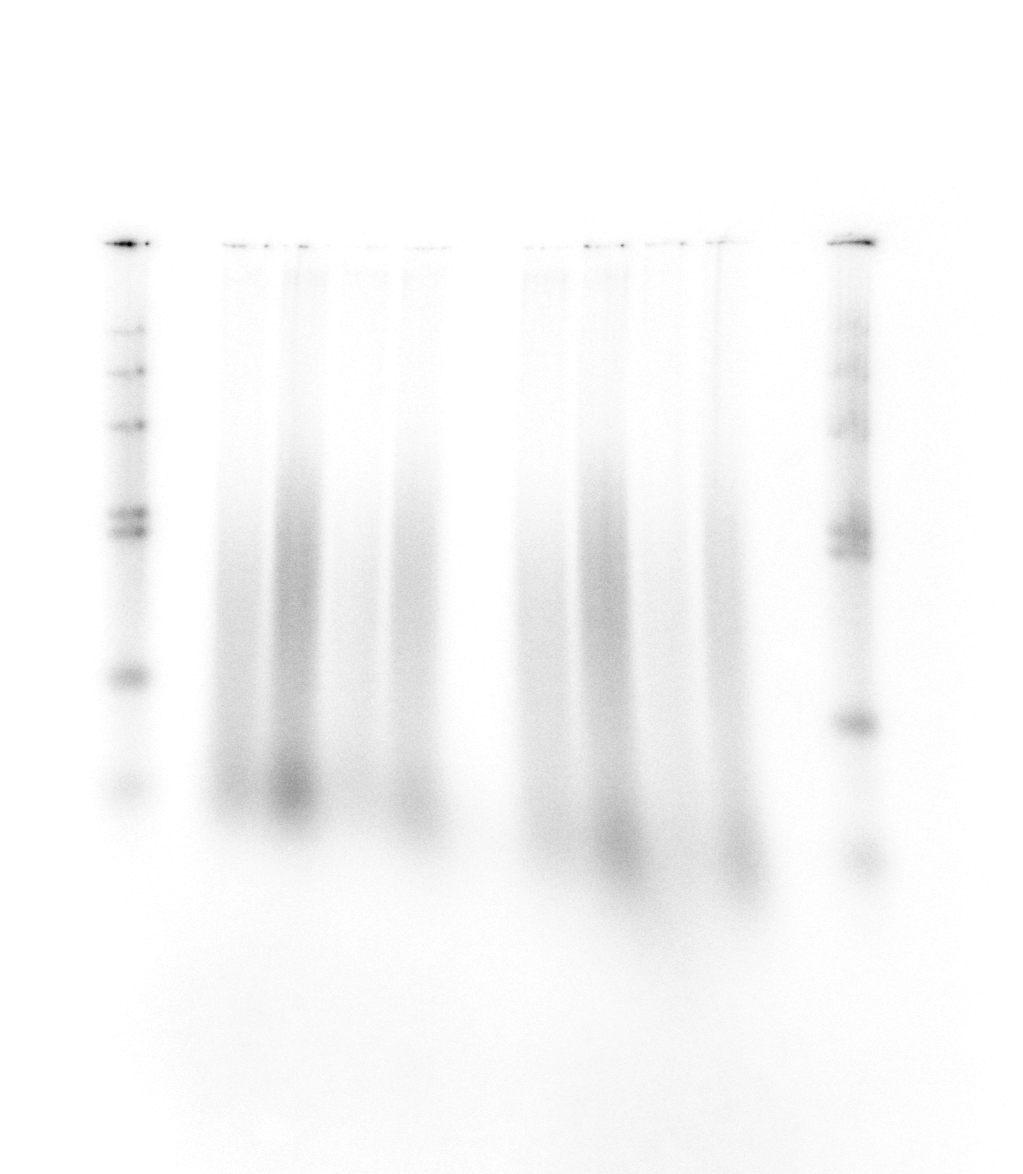

Supplement: Supplementary file 6 — Source data [file 41467_2026_70698_MOESM6_ESM.zip › Uncropped_images/4d.tif]

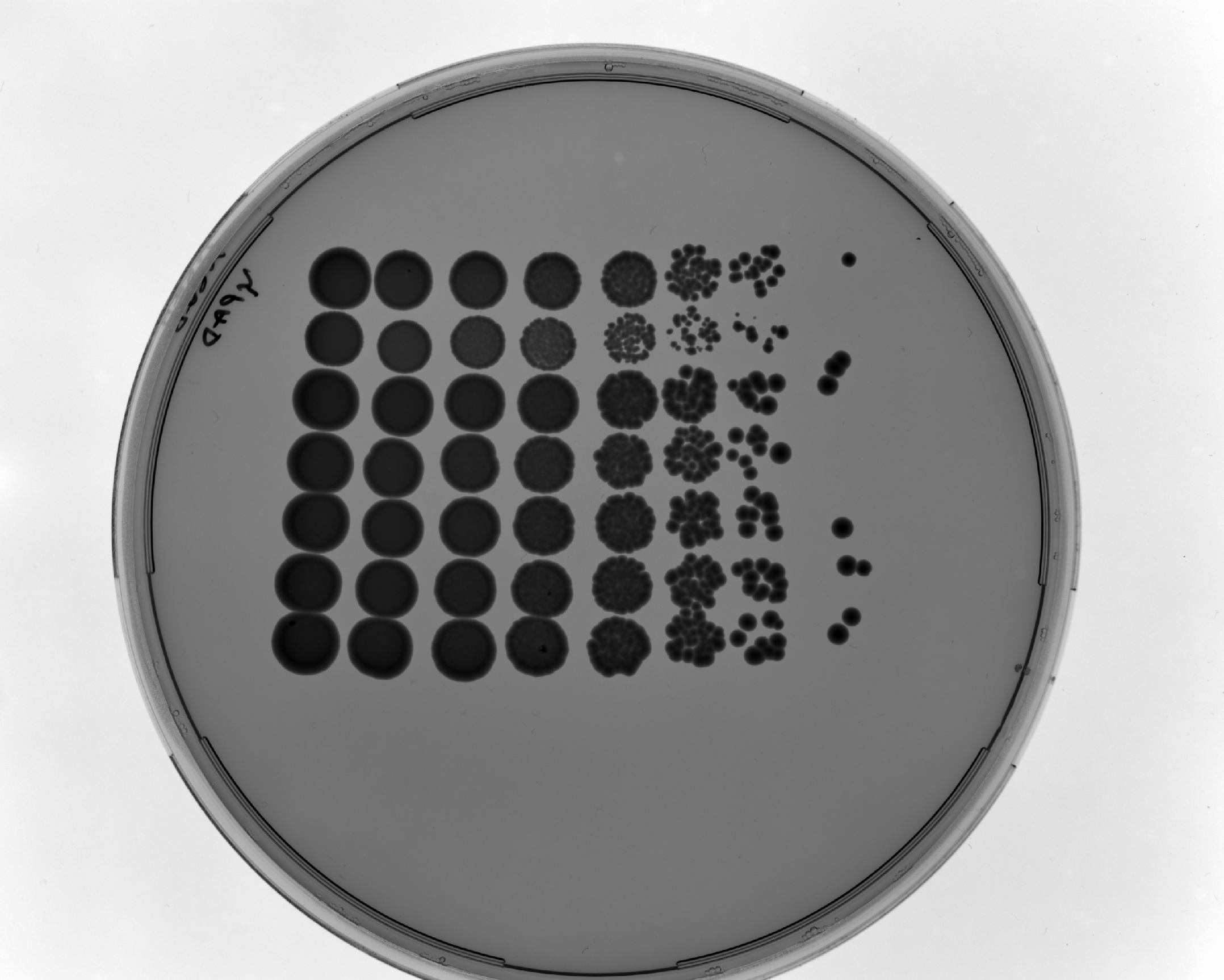

Supplement: Supplementary file 6 — Source data [file 41467_2026_70698_MOESM6_ESM.zip › Uncropped_images/5c.1.tif]

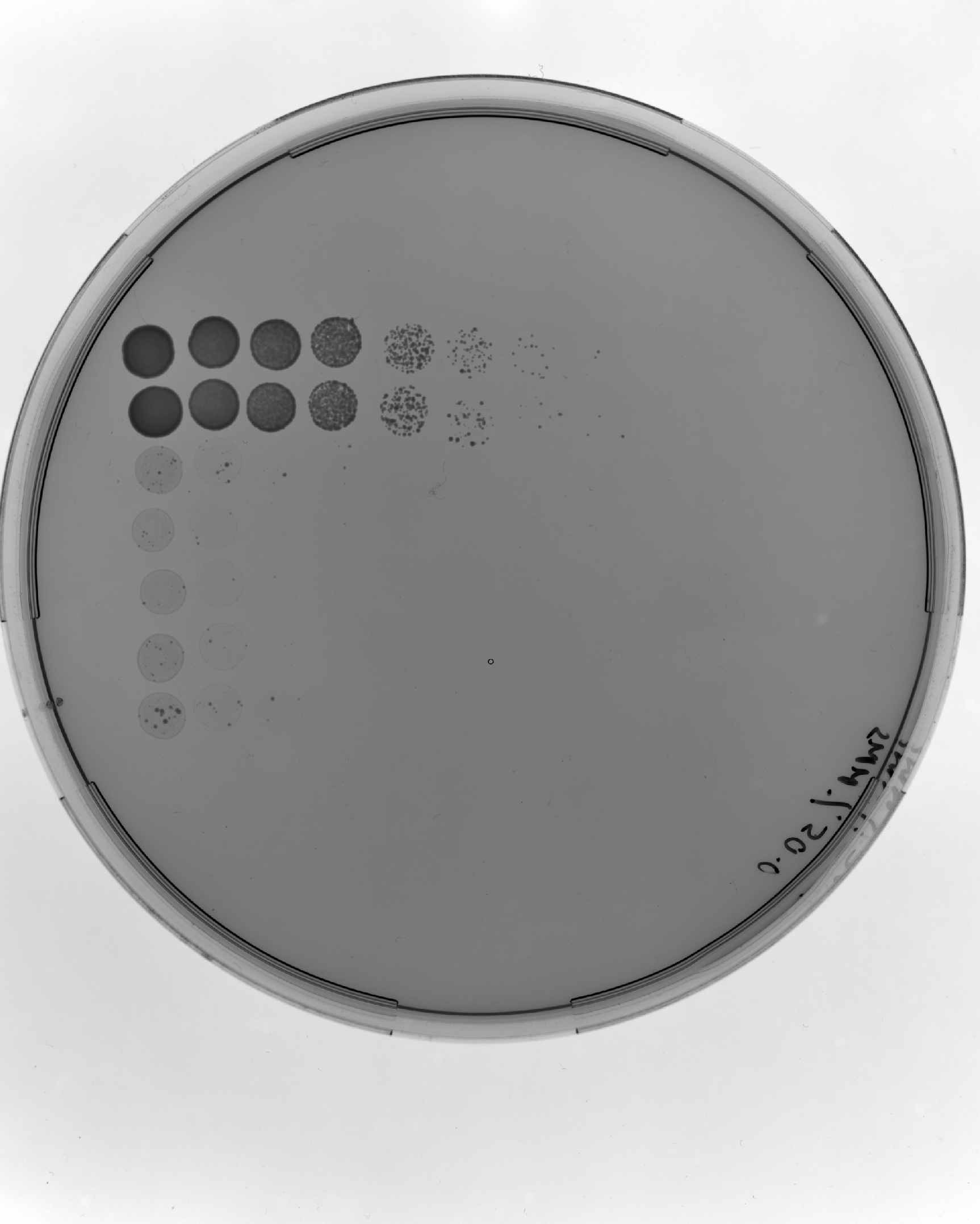

Supplement: Supplementary file 6 — Source data [file 41467_2026_70698_MOESM6_ESM.zip › Uncropped_images/5c.2.tif]

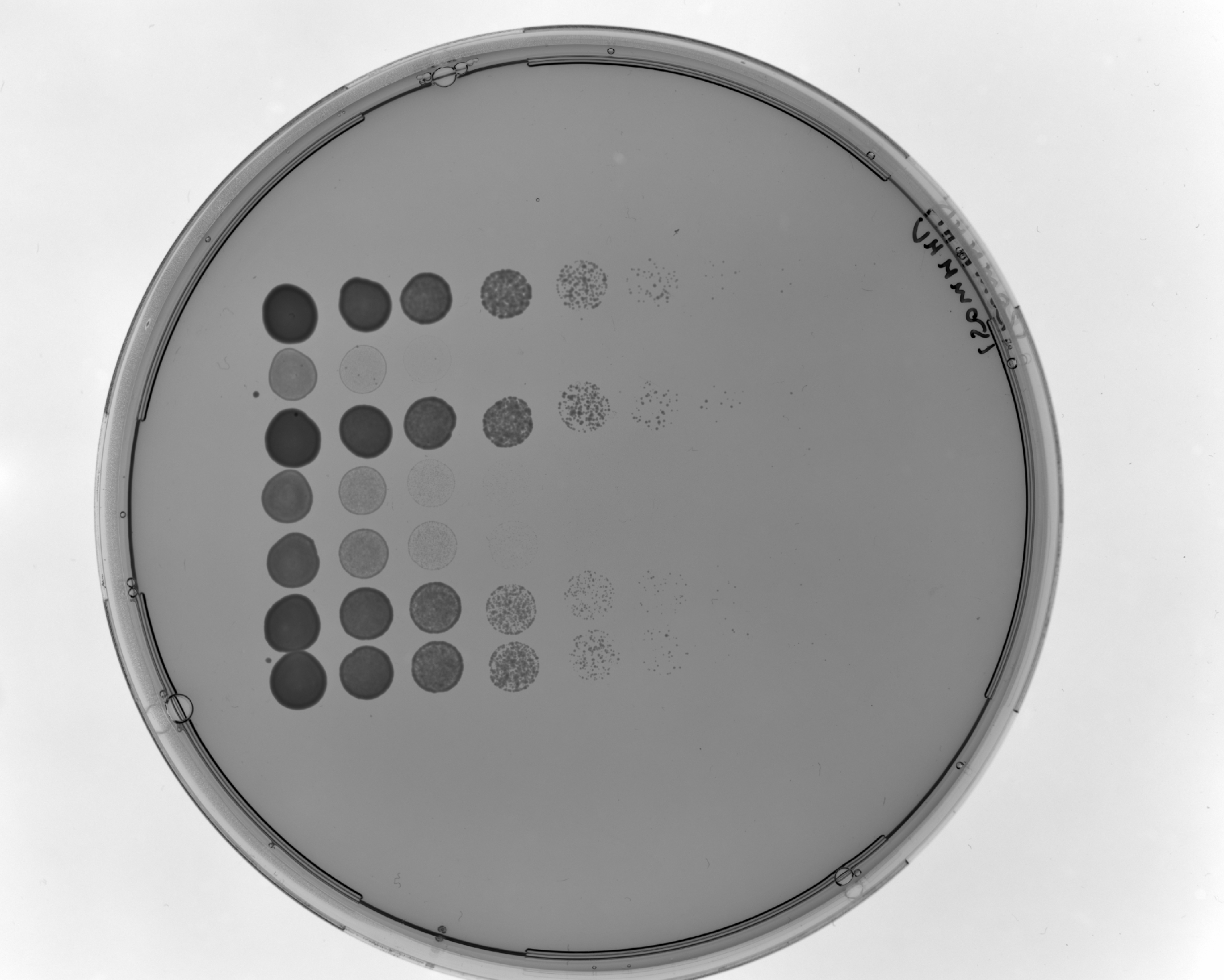

Supplement: Supplementary file 6 — Source data [file 41467_2026_70698_MOESM6_ESM.zip › Uncropped_images/5c.3.tif]

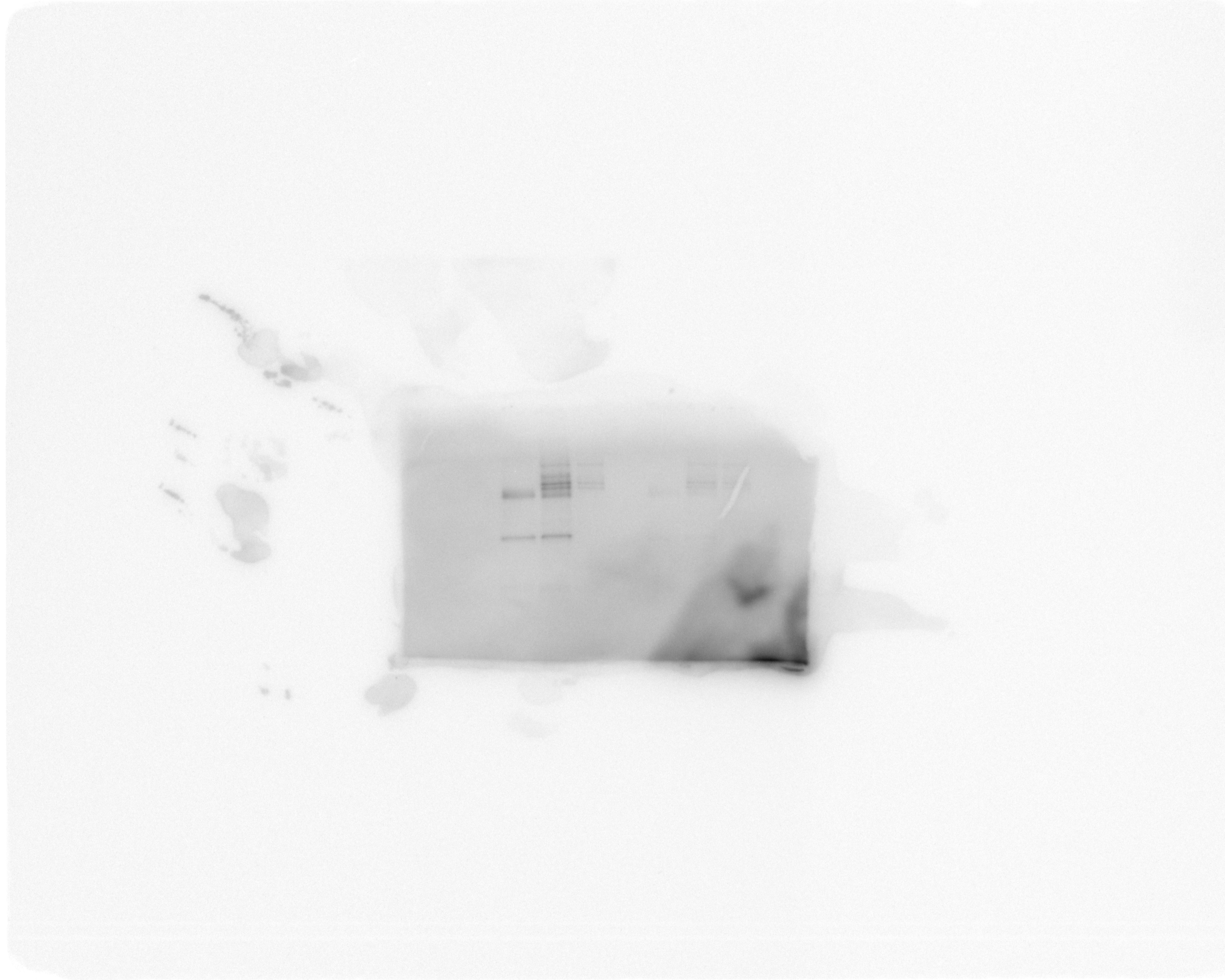

Supplement: Supplementary file 6 — Source data [file 41467_2026_70698_MOESM6_ESM.zip › Uncropped_images/SF3b.tif]

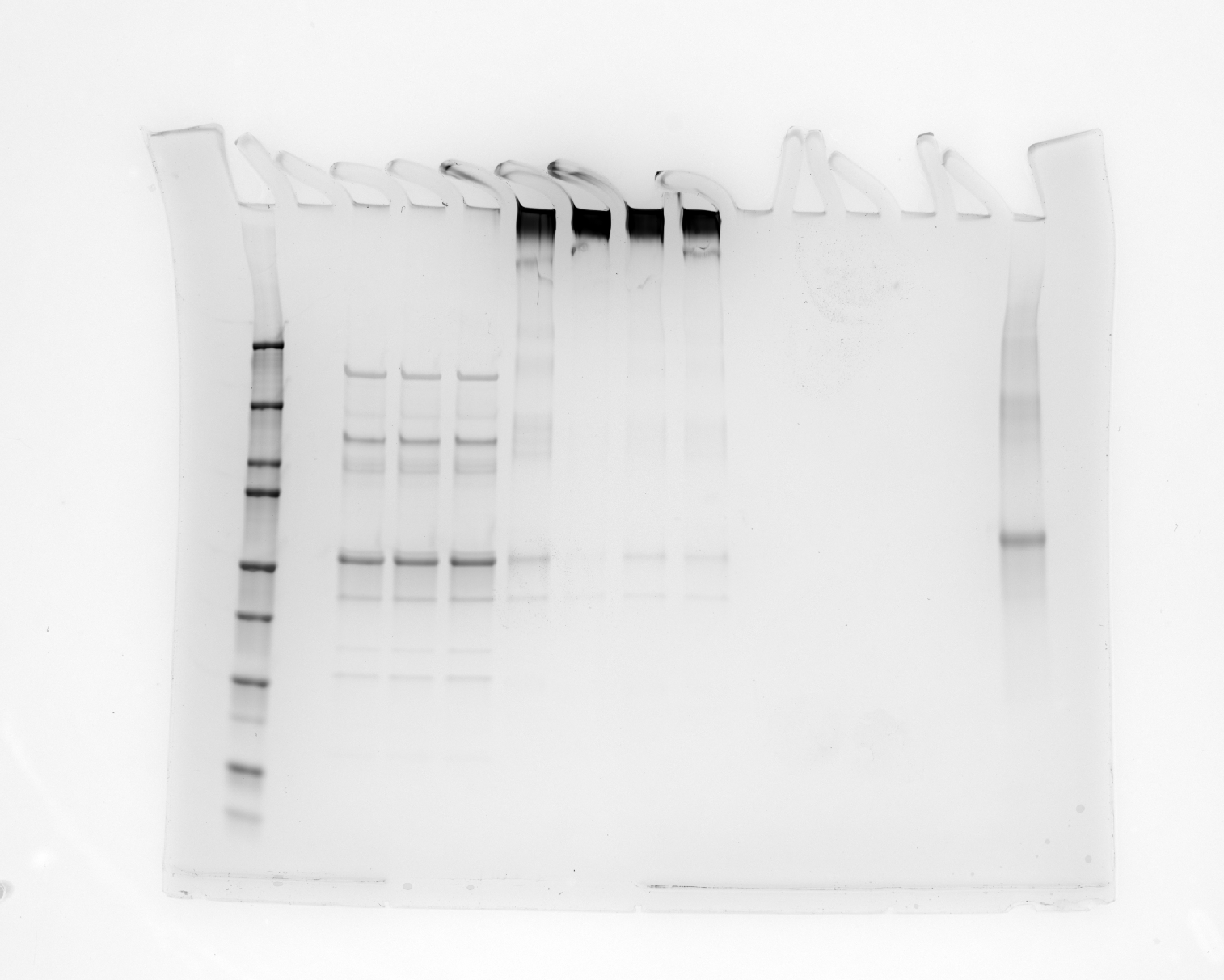

Supplement: Supplementary file 6 — Source data [file 41467_2026_70698_MOESM6_ESM.zip › Uncropped_images/SF4a.1.tif]

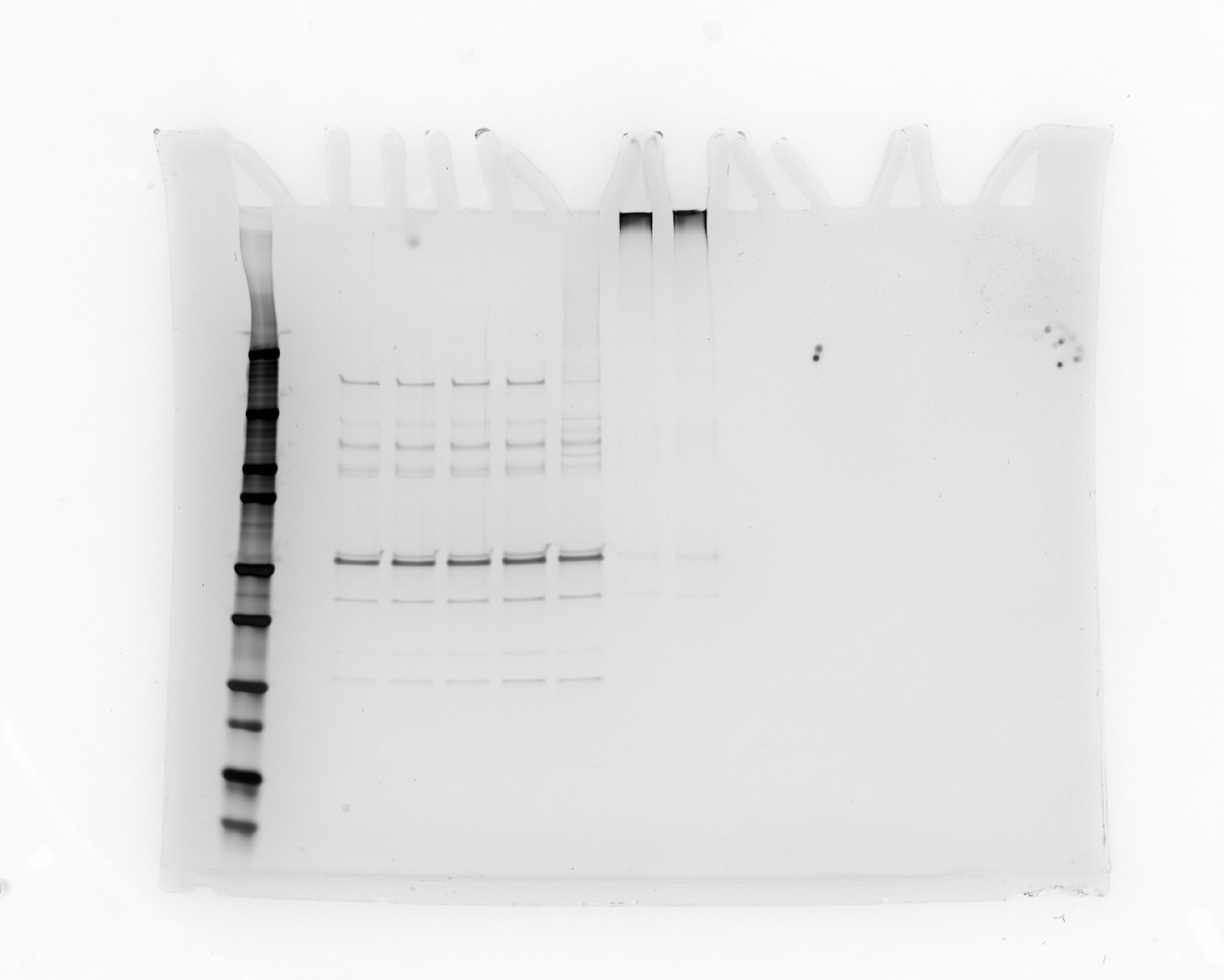

Supplement: Supplementary file 6 — Source data [file 41467_2026_70698_MOESM6_ESM.zip › Uncropped_images/SF4a.2.tif]

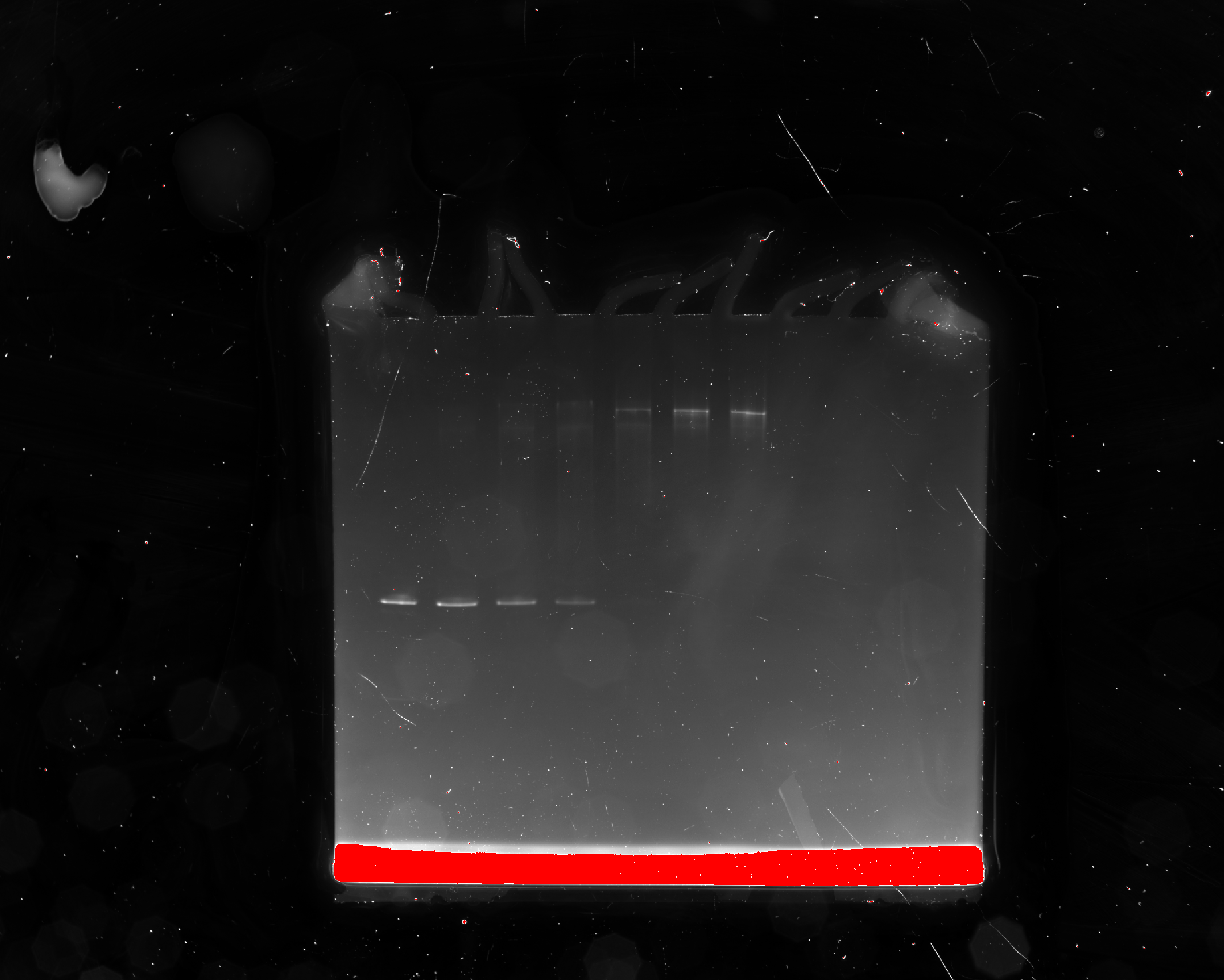

Supplement: Supplementary file 6 — Source data [file 41467_2026_70698_MOESM6_ESM.zip › Uncropped_images/SF6C.1.tif]

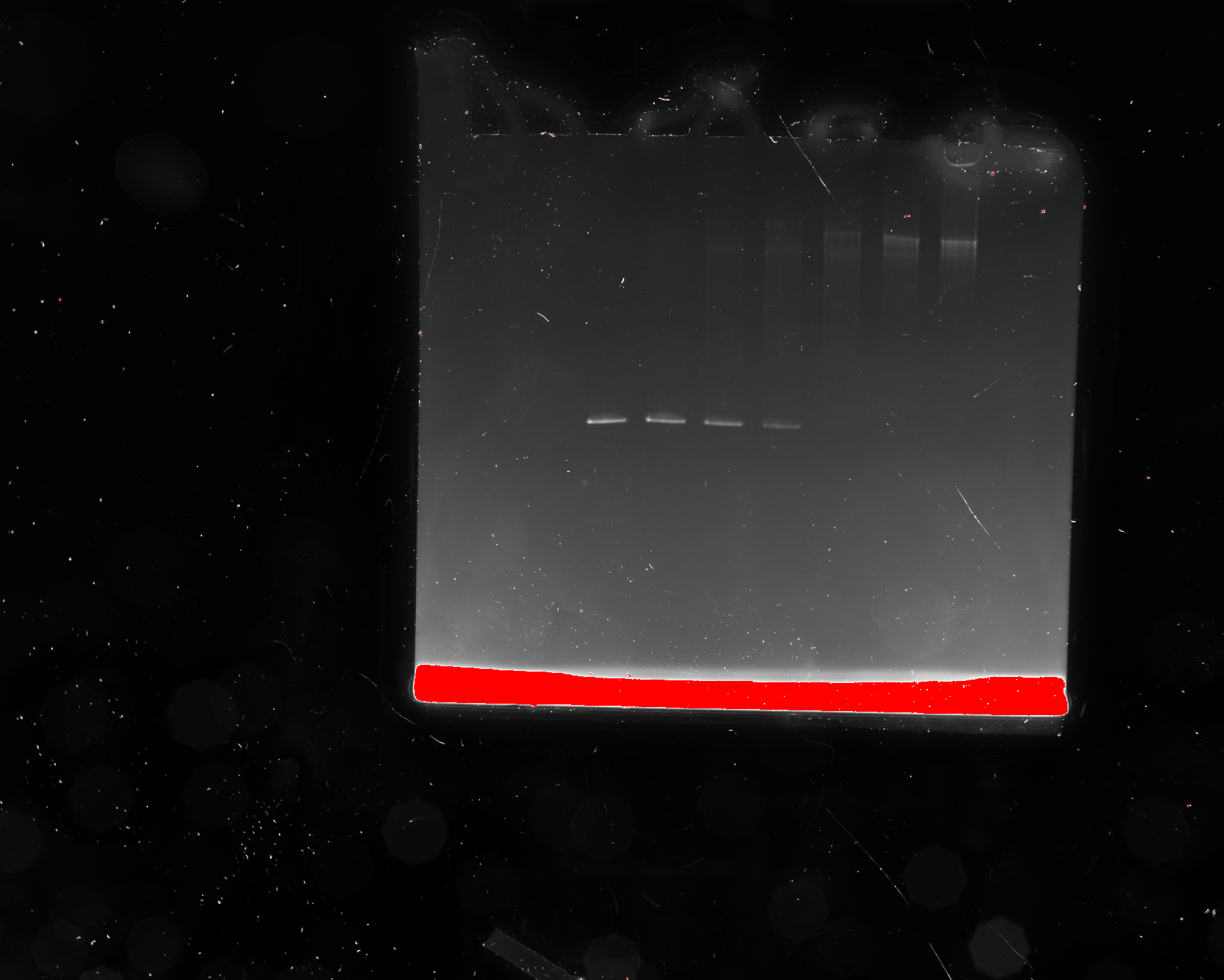

Supplement: Supplementary file 6 — Source data [file 41467_2026_70698_MOESM6_ESM.zip › Uncropped_images/SF6C.2.tif]
